# Heterotrimeric G protein signaling without GPCRs: The *Gα-binding-and-activating* motif

**DOI:** 10.1016/j.jbc.2024.105756

**Published:** 2024-02-15

**Authors:** Mikel Garcia-Marcos

**Affiliations:** 1 Department of Biochemistry & Cell Biology, Chobanian & Avedisian School of Medicine, Boston University, Boston, Massachusetts, USA; 2 Department of Biology, College of Arts & Sciences, Boston University, Boston, Massachusetts, USA

**Keywords:** GPCR, GEF, Girdin, GIV, DAPLE, Calnuc, NUCB2, GBAS-1, PLCD4, CCDC88, RTK, GTPase, integrin, cancer, development, embryo

## Abstract

Heterotrimeric G proteins (Gαβγ) are molecular switches that relay signals from 7-transmembrane receptors located at the cell surface to the cytoplasm. The function of these receptors is so intimately linked to heterotrimeric G proteins that they are named G protein–coupled receptors (GPCRs), showcasing the interdependent nature of this archetypical receptor–transducer axis of transmembrane signaling in eukaryotes. It is generally assumed that activation of heterotrimeric G protein signaling occurs exclusively by the action of GPCRs, but this idea has been challenged by the discovery of alternative mechanisms by which G proteins can propagate signals in the cell. This review will focus on a general principle of G protein signaling that operates without the direct involvement of GPCRs. The mechanism of G protein signaling reviewed here is mediated by a class of G protein regulators defined by containing an evolutionarily conserved sequence named the Gα-binding-and-activating (GBA) motif. Using the best characterized proteins with a GBA motif as examples, Gα-interacting vesicle-associated protein/Girdin and dishevelled-associating protein with a high frequency of leucine residues, this review will cover (i) the mechanisms by which extracellular cues not relayed by GPCRs promote the coupling of GBA motif–containing regulators with G proteins, (ii) the structural and molecular basis for how GBA motifs interact with Gα subunits to facilitate signaling, (iii) the relevance of this mechanism in different cellular and pathological processes, including cancer and birth defects, and (iv) strategies to manipulate GBA-G protein coupling for experimental therapeutics purposes, including the development of rationally engineered proteins and chemical probes.

## Canonical activation of G protein signaling by GPCRs

In an interview after receiving the Nobel Prize for the discovery and characterization of heterotrimeric G proteins, Al Gilman humorously said that these G proteins are involved in “*everything from sex in yeast to cognition in humans*” (1). This is not far from reality given that they are key mediators of intercellular communication in numerous contexts (2, 3) and across different species (4, 5). Their best established role is to serve as cytoplasmic transducers of signals generated by cell surface receptors characterized by the presence of seven transmembrane helices, which are generically named G protein–coupled receptors (GPCRs) (2, 3). This signaling paradigm has proven to be an evolutionary success of unparalleled versatility; it is used across eukaryotes to sense and respond to an enormous number of extracellular cues, including light, odors, pressure, tastes, pH, metabolites, neurotransmitters, and hormones, among others (4, 5, 6). In humans, this mechanism is also targeted by more than one-third of clinically approved drugs, highlighting its broad biomedical relevance (7, 8, 9).

Heterotrimeric G proteins are named after their composition of three different types of subunits: Gα, Gβ, and Gγ subunits. Gβ and Gγ function as obligatory Gβγ dimers in cells, and Gα′s are the nucleotide binding subunits that serve as ON/OFF switches. When loaded with GDP, Gα subunits bind tightly to Gβγ and the trimeric complex is silent for signaling. Activated GPCRs recognize this resting trimeric complex as the substrate for their guanine-nucleotide exchange factor (GEF) activity—that is, they promote the release of GDP from Gα and the subsequent spontaneous loading of GTP present at high concentration in the cytoplasm. Once turned on by GTP binding, Gα subunits dissociate not only from Gβγ but also from receptors, which ensures that signals propagate unidirectionally toward modulation of downstream G protein targets. Active Gα and Gβγ modulate specific effectors. Depending on the structural and functional similarity of Gα subunits, they are classified in four families (G_s_, G_i/o_, G_q/11_, G_12/13_), which engage different effectors (2). For example, Gαs activates, whereas Gαi inhibits, adenylyl cyclases, and Gαq activates phospholipase C enzymes, whereas Gα13 triggers the action of a family of RhoGEFs. The duration of signaling is determined by how long Gα remains bound to GTP, which is in turn regulated by their own GTPase activity. Once GTP is hydrolyzed, GDP-bound Gα can reassociate with Gβγ and become the starting point for a new round of activation by GPCRs. While there are exceptions for these general rules, like rearrangement of Gα and Gβγ instead of physical dissociation or the formation of unproductive GPCR–G protein complexes (10, 11, 12, 13), the mechanism described above is considered the canonical “G protein cycle” upon GPCR regulation.

## Historical perspective on nonreceptor regulators of G protein signaling

**Figure 1:**
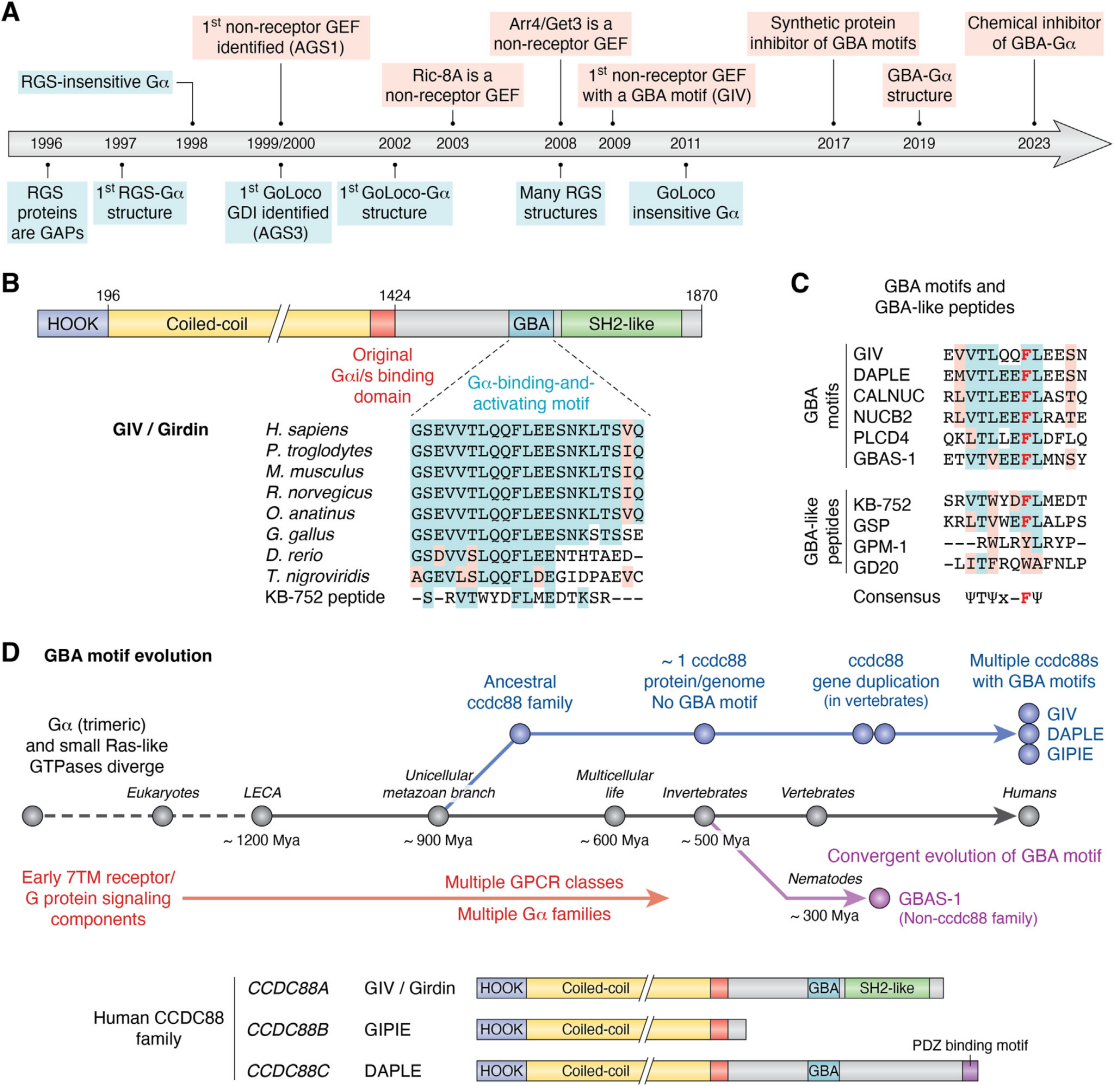
**Identification of an evolutionarily conserved mechanism of G protein activation by proteins with a GBA motif.***A*, timeline of some discoveries related to non-GPCR regulators of G proteins. Nonreceptor GEFs were identified and characterized later than GAPs and GDIs. *B*, GIV/Girdin contains and evolutionarily conserved motif required for the activation of G protein signaling. *C*, alignment of GBA motifs identified in proteins and of GBA-like peptides. *D*, GBA motif in evolution. CCDC88 proteins acquired a GBA motif upon gene duplication in the transition from invertebrates to vertebrates, whereas the GBA motif–containing protein GBAS-1 appeared earlier by convergent evolution from a distinct lineage in nematodes. GBA, Gα-binding-and-activating; GEF, guanine-nucleotide exchange factor; GIV, Gα-interacting vesicle-associated protein; GPCR, G protein–coupled receptor.

Discoveries over the last decades have progressively revealed increasing complexity in the mechanisms that control heterotrimeric G protein signaling, expanding them beyond the canonical G protein cycle mentioned above. It has become clear that in addition to GPCRs, G protein signaling is controlled by numerous cytoplasmic, nonreceptor proteins that bind to and regulate the activity of Gα subunits (14, 15, 16, 17, 18, 19, 20, 21, 22, 23, 24, 25, 26). By affecting how Gα binds and hydrolyzes nucleotides, these regulators have profound effects on the timing and strength of signaling (14, 15, 16, 17, 19, 20, 21, 22, 23, 24, 25, 26). As it happens for virtually any other type of G protein (27), these regulators can be broadly divided into groups based on the biochemical activity they exert on the nucleotide handling functions of Gα—for example, they can promote GDP/GTP exchange by acting as GEFs (28, 29, 30, 31, 32, 33), much like GPCR do, or prevent GDP dissociation serving as guanine-nucleotide dissociation inhibitors (GDIs) (14, 34, 35, 36, 37, 38, 39, 40), or enhance the intrinsic GTP hydrolysis by G proteins when they are GTPase-accelerating proteins (GAPs) (20, 41, 42, 43). Of these classes of regulators, the best characterized are GAPs and GDIs, whereas the characterization and understanding of nonreceptor GEFs has lagged behind. A factor that might have contributed to a better understanding of GAPs and GDIs is that the signature domains or motifs responsible for their biochemical activities were identified early on, allowing more systematic efforts for their structural and functional characterization (Fig. 1*A*). For example, although some G protein effectors like PLCβ isoforms have GAP activity (44), an entire family of GAPs named regulators of G protein signaling (RGS) proteins was discovered based on the conservation of the “RGS box” domain of ∼120 amino acids (19, 20, 22, 24, 25, 45, 46, 47, 48, 49). Similarly, a family of GDIs was grouped together based on the presence of a signature sequence of ∼20 to 30 amino acids named the GoLoco motif, also known as the G protein regulatory (GPR) motif (14, 34, 36, 37, 39, 40). For both RGS and GoLoco motifs, the structural basis for their action of Gα subunits was elucidated soon after their initial discovery (50, 51), which spurred further functional studies and attempts to pharmacologically target them (52). For example, these structural characterizations contributed, at least in part, to the development of RGS-insensitive and GoLoco-insensitive G proteins that have been leveraged in cellular and animal models to elucidate the physiological consequences of GAP- or GDI-mediated regulation of G protein signaling (53, 54, 55, 56).

While research on GAP and GDI families unified by the presence of shared functional modules advanced rapidly, nonreceptor GEFs remained as a heterogeneous group of proteins without a signature domain or sequence (Fig. 1*A*). Examples include proteins like activator of G protein signaling 1 (AGS1)/DEXRAS (31), Ric-8A (57), Ric-8B (58), Arr4/Get3 (32), or CSPα (59), among some others (14). Some of these were even discovered at the same time as GoLoco motif–containing GDIs as part of genetic screens for the identification of “AGS” (18, 60), yet progress in characterizing them lagged behind because of the lack of tools. The paucity of tools is exemplified by the fact that no GEF-deficient mutants could be rationally designed to unequivocally link the biological functions of these proteins to their GEF activity instead of to other functions these proteins may have. For example, AGS1/DEXRAS is itself a Ras-like G protein that modulates calcium and nitric oxide signaling in addition to heterotrimeric G proteins (61), and Arr4/Get3 has also been described as a critical component of a complex that mediates membrane protein targeting to and insertion into the endoplasmic reticulum (62). However, in 2009, the protein named Gα-interacting vesicle-associated protein (GIV), also known as Girdin and encoded by the human gene *CCDC88A*, was described as the first example of a nonreceptor GEF for Gα for which both the biochemical activity *in vitro* and the stimulation of G protein signaling in cells could be ascribed to a defined sequence motif (30). This review will focus on the developments that followed this seminal discovery over the last ∼15 years, which resulted in the identification and characterization of a class of G protein signaling activators with GEF activity. The presence of a defined motif, named the *Gα-binding-and-activating* (GBA) motif, has enabled the identification of additional members of the same class of regulators, dissecting their structural basis and mechanisms of action in cells, defining the role of this type of G protein regulation in physiology and disease, and the development of tools to modulate their activity for research purposes or in models of experimental therapeutics, including genetically encoded proteins and a small molecule inhibitor. It is important to note that not all nonreceptor GEFs contain a GBA motif and that the GBA motif might regulate G proteins through mechanisms that are independent of its GEF activity (see below). Nevertheless, the focus of this review on GBA motif–containing proteins is warranted because it provides a prime example of detailed understanding of activation of G protein signaling without GPCRs across scales of biological organization, from structure to (patho)physiology, and also because it illustrates new potential avenues for therapeutic targeting.

## Discovery of an evolutionarily conserved mechanism of GPCR-independent activation of heterotrimeric G proteins

### Identification of a GPR motif in GIV

As mentioned above, the dogma that activation of heterotrimeric G protein signaling was an exclusive task of GPCRs had been challenged, at least in part, by the identification of cytoplasmic proteins with GEF activity. Although GIV was originally identified as a Gαi and Gαs-binding protein (63), it was not until later that it was found that it had GEF activity conferred by a defined motif. This came to light as a result of efforts to map the Gαi-binding site on GIV. Despite original findings that GIV-bound Gαi through a region close to the C-terminal end of its central coiled-coiled domain (Fig. 1*B*), it was found that binding was much more robust with the C-terminal region of the protein (30). Moreover, binding to this C-terminal region was selective for inactive, GDP-bound Gαi, a feature shared with other G protein regulators including GEFs. The hypothesis that GIV might serve as a GEF was solidified by the realization that an evolutionarily conserved stretch of ∼30 amino acids in the C-terminal G protein–binding region also had similarity with a synthetic GEF peptide named KB-752 (64) (Fig. 1*B*). This peptide had been original identified as part of an unbiased screen for state-selective G protein binders unrelated to GIV or non-GPCR activators (64). Yet, much like GIV, KB-752 was a Gα-GDP selective binder with GEF activity for Gαi subunits *in vitro* (64). Moreover, the original discovery of the KB-752 peptide was accompanied by the elucidation of its structure in complex with Gαi by X-ray crystallography (64). By modeling based on the KB-752/Gαi structure, specific mutations were designed in the putative GEF motif of GIV to successfully disrupt its binding to Gαi (30). Using “gold standard” *in vitro* enzymatic assays, it was confirmed that GIV had GEF activity toward Gαi and that this activity was ablated when the putative GEF motif was mutated (30, 65). These results demonstrated that the evolutionarily conserved sequence in GIV with similarity to the KB-752 peptide, which was later named the GBA motif, was necessary to confer GEF activity to GIV. As described in subsequent sections, the GBA motif of GIV was also required to promote G protein signaling in cells.

### Discovery of a class of G protein regulators that share a GBA motif

The discovery of the first “GEF motif” in the protein GIV raised the interesting question of whether there were other proteins with equivalent GPR activity due to the presence of a similar motif. In other words: does the GBA motif define a class of G protein regulators? Initial efforts in this regard were not systematic and relied instead on candidate approaches and educated guesses. First, attention was put on other G protein interactors that were poorly characterized at the time, like Calnuc (also known as NUCB1), which had been identified along with GIV in yeast two-hybrid screens using Gαi3 as a bait (66, 67). Both Calnuc and the closely related protein NUCB2 were found to contain a sequence similar to the GBA motif (68) (Fig. 1*C*). Calnuc and NUCB2 bound to Gαi-GDP but not to Gαi-GTP and promoted nucleotide exchange *in vitro*, much like GIV. Both G protein binding and the GEF activity of Calnuc and NUCB2 were ablated upon mutation of the GBA motif, providing the first example of functional conservation across different proteins based on the presence of this signature sequence of GIV (68). An interesting feature of Calnuc and NUCB2 is that their respective GBA motifs overlap with the sequence of their calcium-binding EF-hands. Upon Ca^2+^ binding, these EF-hands adopt a conformation that buries the GBA motif and makes it inaccessible for G proteins. However, the relatively low affinity of Calnuc and NUCB2 for Ca^2+^ (69) indicate that in resting cells, they would engage G proteins through their GBA motif because the concentration of cytosolic Ca^2+^ is too low to bind to the EF-hands (∼50–100 nM). In contrast, when a stimulus raises the concentration of cytosolic Ca^2+^ (*e.g.*, ∼1 μM or higher), G protein binding to Calnuc and NUCB2 is blocked (68), suggesting that the GPR activity mediated by their GBA motifs is modulated by changes in the cytosolic concentration of Ca^2+^. Nevertheless, despite hints on the interplay between Calnuc and G proteins in cells (70), the specific role of Calnuc or NUCB2 in regulating G protein signaling in cells remains undefined.

Another putative GBA motif–containing protein was identified by exploring candidates evolutionarily related to GIV. In vertebrates, GIV belongs to a small family of three proteins encoded by *CCDC88* genes: GIV (*CCDC88A*), GRP78-interacting protein induced by ER stress, encoded by *CCDC88B*, and dishevelled-associating protein with a high frequency of leucine residues (DAPLE), encoded by *CCDC88C* (71). These proteins share higher homology in their N-terminal region and the long coiled-coiled but diverge in the C-terminal region where the GBA motif was found in GIV (71). However, close inspection of the C-terminal region of DAPLE revealed the presence of cryptic GBA motif similar to that originally identified in GIV and conserved across species (72) (Fig. 1*C*). DAPLE recapitulated all the GPR features previously observed for GIV: it bound state selectively to inactive Gαi and promoted nucleotide exchange *via* its GBA motif (72).

Given the predictive power of sequence similarity with the GBA motif to identify G protein regulators with GEF activity, a more systematic effort was envisioned by Maziarz and colleagues (73). They established a discovery pipeline in which candidate sequences identified bioinformatically were screened for binding to Gαi as isolated sequences using peptide arrays and subsequently tested in a functional assay of G protein signaling in yeast when expressed in the context of the proteins they belonged to. Candidates that passed these filters were eventually validated with *in vitro* biochemical assays and experiments in mammalian cells, which revealed that PLCδ4b acted as a nonreceptor GEF for Gαi *via* a GBA motif (73) (Fig. 1*C*). Interestingly, the yeast-based assay used in this pipeline was the same that had been previously used to discover AGS proteins like AGS1 and AGS3 (18, 60) and gave robust responses with various GBA motif–containing proteins, suggesting that the latter could also be classified as a subgroup of AGS proteins.

### Evolutionary conservation of GBA motif–mediated G protein regulation

The observation that GIV and DAPLE, but not GRP78-interacting protein induced by ER stress, contains a GBA motif prompted a further investigation of the evolutionary origins of the GBA motif as a GPR sequence. While there are typically three members of the *CCDC88* family in vertebrate animal species and at least one of them has a GBA motif, invertebrate animal species only have one *CCDC88* gene that does not bear a GBA motif (28, 74) (Fig. 1*D*). Furthermore, vertebrate proteins encoded by *CCDC88* genes all share an N-terminal region and coiled-coil region homologous to that of invertebrate *CCDC88*, but only GIV and DAPLE have a nonconserved, extended C-terminal region where the short sequence corresponding to the GBA motif is one of the few fragments with similarity between the two proteins (71, 72, 75). It appears as if the expansion of the ancestral *CCDC88* gene in invertebrates into the multiple *CCDC88* genes of vertebrates was accompanied, at least in some cases, by the acquisition of a GBA motif that was preserved under evolutionary selective pressure. This led to the question of whether other invertebrate proteins unrelated to *CCDC88* genes could have independently acquired a GBA motif to regulate G proteins. The uncharacterized protein F59H5.1 was identified as the top candidate from a bioinformatics search of GBA-like sequences in the worm *Caenorhabditis elegans*, which was subsequently named GBAS-1, for GBA and SPK domain containin-1 (74). GBAS-1 not only had GEF activity for its cognate nematode G protein GOA-1 but was also coexpressed with the G protein in a variety of cells in living nematodes, and loss of GBAS-1 led to phenotypes in behaviors controlled by GOA-1 (74). Moreover, GBAS-1 could also bind and activate mammalian G proteins, which revealed the existence of evolutionarily conserved determinants of the GBA-G protein interface. As opposed to *CCDC88*, GBAS-1 had no homologs in vertebrates and only some closely related nematode species had GBAS-1 homologs with a GBA motif (74). The convergent evolution of a GBA motif in highly divergent proteins present exclusively in invertebrates asserts the modular basis of a GPCR-independent mechanism of G protein regulation that appeared at least 300 million years ago in metazoans (28).

## Structural and molecular basis for GBA motif–mediated regulation of G protein signaling

### Early observations based on homology modeling

**Figure 2:**
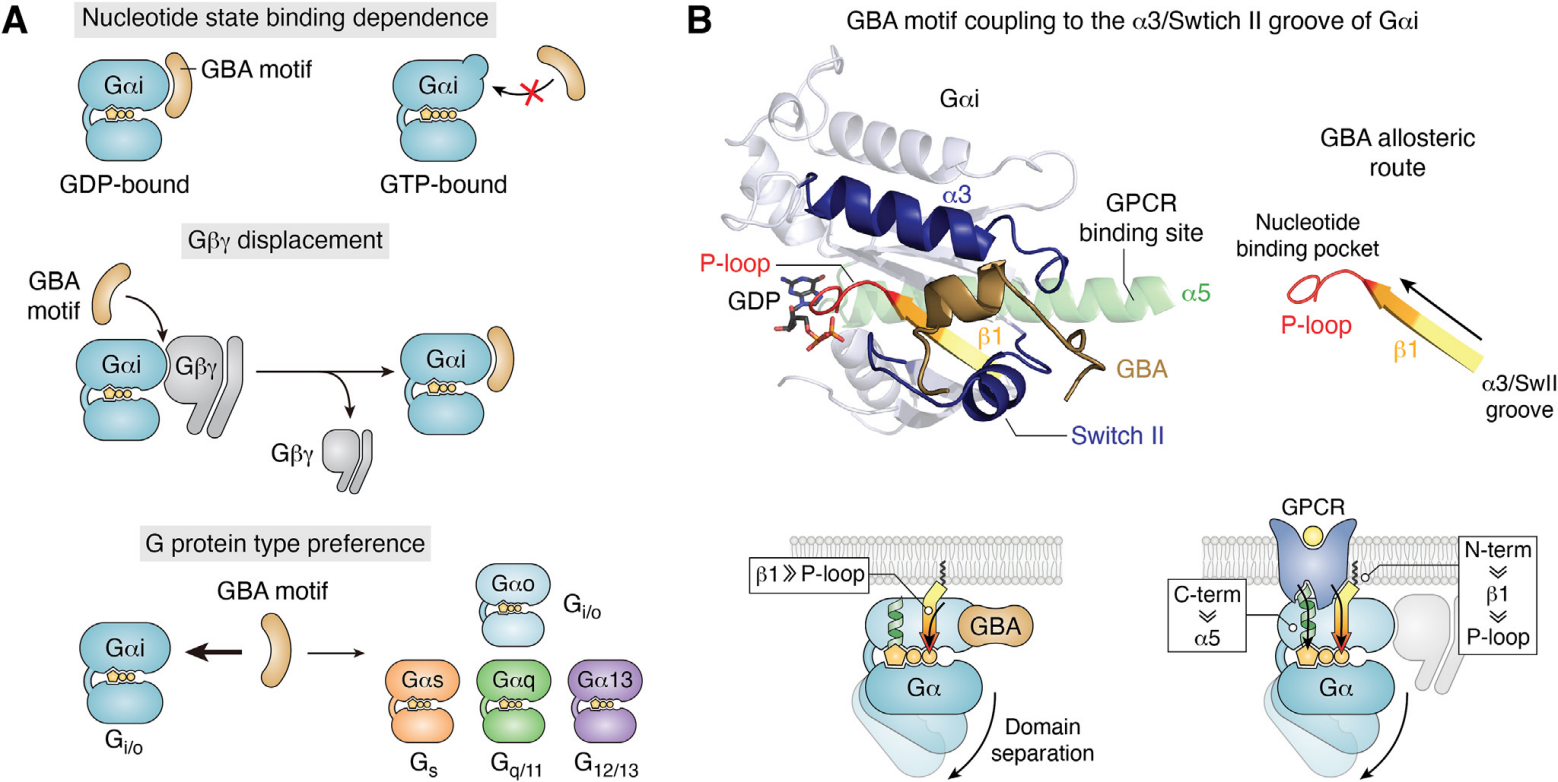
**Molecular and structural basis for the action of GBA motifs on G proteins.***A*, specific features of the interaction between GBA motifs and Gαi subunits. GBA motif binds preferentially to inactive over active Gα subunits and can displace Gβγ from preformed heterotrimers in the absence of nucleotide exchange. GBA motifs bind primarily to Gai isoforms, whereas it binds weakly to other members of the G_i/o_ family or of other G protein families. *B*, GBA motifs bind to the groove formed between the α3 helix and the switch II in Gαi, which is communicated allosterically to the P-loop in the nucleotide binding pocket *via* the β1 strand. The binding site of GBA motifs of Gα does not overlap with that of GPCRs, but the allosteric routes that communicate with the nucleotide binding pocket might be partially shared between GPCRs and GBA motifs. GBA, *Gα-binding-and-activating*; GIV, Gα-interacting vesicle-associated protein; GPCR, G protein–coupled receptor.

Insights into the structural basis for G protein regulation by GBA motifs were initially gained from homology models guided by the structure of the GEF peptide KB-752 in complex with Gαi (64) (Fig. 2). Based on this, GBA motifs were predicted to engage a groove formed by the α3 helix and the switch II region of the G protein, which was confirmed using site-directed mutagenesis of amino acids on both sides of the protein–protein interface (30, 65, 68, 72, 76). Binding to this region explains well the selectivity of GBA motifs for GDP-bound Gαi, given that the switch II is one of the regions in the G protein that changes conformation drastically upon GTP binding. In fact, the groove where GBA motifs dock is largely occluded by a helical structure formed by the switch II in Gαi-GTP (Fig. 2). A second insight gained from these early observations was that the binding site for GBA motifs on Gαi-GDP overlaps with the binding site for Gβγ (Fig. 2). It was indeed demonstrated that GBA motifs compete with Gβγ for binding to Gαi-GDP and that they exert their GEF activity when Gα-GDP is in its monomeric form or after they have caused the dissociation of preformed Gα-Gβγ trimers (30, 72, 73).

The early identification of the α3 helix/switch II groove as the binding site for GBA motifs also shed light onto the basis for their G protein selectivity, even within the G_i/o_ family. For example, Gαo bound GBA motifs of GIV, DAPLE, Calnuc, or NUCB2 much less than Gαi isoforms, which could be ascribed to differences in two amino acids in the α3 helix/switch II region (K248 and W258 of Gαi3) (65, 68, 72, 74) (Fig. 2). Differences in this region across G proteins also help explain their weaker binding of GBA motifs to G proteins that are not Gαi in general, although binding of some GBA motifs to other Gα proteins has been reported. For example, DAPLE can bind Gαs and Gαq (77) and certain phosphoforms of GIV also bind to Gαs (78). Interestingly, when DAPLE or GIV bind to non-G_i/o_ family G proteins, they work as GDIs instead of GEFs (77, 78). These findings prompted the terminology guanine-nucleotide exchange modulator to broadly define the class of G protein regulators that contain a GBA motif (79). However, the direct biological consequences of the reported GDI activities remain undefined, and, as explained below, even the specific role of the GEF activity of GBA motifs on Gαi (*i.e.*, acceleration of GTP loading) in cell signaling still requires further investigation.

### Structural basis of G protein binding and allosteric regulation of nucleotide binding

Further studies combining structure modeling, NMR, and systematic mutagenesis of both the GBA motif and Gαi not only confirmed that the binding GBA motifs to G proteins is very different from that observed for GPCRs, the canonical GEFs, but also the allosteric mechanisms that influence nucleotide exchange are different between receptor and nonreceptor GEFs (80). GPCRs bind primarily to the C- terminus of Gα subunits, with potentially some contribution of other elements like the αN-β1 hinge, to induce conformational changes in the nucleotide-binding pocket allosterically (81). In contrast, GBA motifs appeared to bind primarily to the α3 helix/switch II groove of Gαi without making direct contact with the nucleotide-binding pocket and without overlapping with GPCR binding sites (Fig. 2), which was subsequently confirmed through the elucidation of the structure of Gαi3 bound to GIV’s GBA motif *via* X-ray crystallography (82). While GBA binding led to clear perturbations of nucleotide binding elements as observed by NMR, there were remarkable differences with the perturbations induced GPCRs in the nucleotide-binding pocket (28, 80, 83). The most notable one is that GPCRs induce changes in all nucleotide binding elements of the G protein (84, 85), whereas GBA motifs only perturbed the phosphate binding elements P-loop and switch I, which are likely propagated from the α3 helix/switch II groove *via* the β1 strand (80) (Fig. 2). Overall these observations indicated that GBA motifs and GPCRs promote nucleotide exchange through fundamentally different mechanisms. The direct substrate for the GEF activity of GBA motifs are Gα monomers, whereas GPCRs work on heterotrimers, and the perturbations in the nucleotide-binding pocket appear to follow different, albeit partially overlapping, allosteric routes (80, 83). For GBA motifs, the perturbations in the nucleotide-binding pocket appear to be less pronounced than with GPCRs, which is also in agreement with their weaker GEF activity.

### Do GBA motifs promote Gα-GTP and/or Gβγ signaling in cells?

A question that arose early on from the characterization of the structural and molecular mechanism of action of GBA motifs on G proteins was about the generation of signaling species in cells (30). Heterotrimeric G proteins can give rise to two active signaling species: GTP-bound Gα and free Gβγ. As mentioned above, GBA motifs not only display GEF activity on Gαi *in vitro*, indicating that they can generate Gα-GTP, but can also cause the dissociation of Gβγ from heterotrimers even in the absence of nucleotide exchange (30, 72). From these observations *in vitro*, the question that followed was about the relative contribution of Gαi-GTP and free Gβγ to GBA motif–mediated signaling in cells—that is, is the signaling triggered by GBA motifs in cells caused by Gαi-GTP or by the release of Gβγ? Moreover, since Gαi-GTP cannot bind Gβγ, how much of GBA-dependent Gβγ signaling arises from the generation of Gαi-GTP *versus* physical, competitive displacement in the absence of nucleotide exchange? Answering these questions was made possible by new bioluminescence resonance energy transfer–based biosensor tools that directly measure the formation of Gα-GTP in cells (86), which were leveraged in combination with a chemogenetic approach to precisely control the action of GBA motifs on G proteins in cells (87). Real-time bioluminescence resonance energy transfer measurements revealed that formation of Gαi-GTP in cells by GBA motifs was undetectable, whereas they led to formation of free Gβγ at levels equivalent to those observed upon GPCR stimulation (87). Consistent with this observation, GBA motif–meditated cAMP inhibition was also completely suppressed by Gβγ scavenging, indicating that the observed modulation of the levels of this second messenger was not a consequence of Gαi-GTP formation as previously assumed (73). These results indicating that Gβγ is the primary species responsible for GBA-mediated signaling should be taken with caution given that the chemogenetic system used to draw these conclusions might not fully recapitulate the mechanisms by which GBA motifs operate under native, receptor-initiated conditions. However, the vast majority of receptor-initiated responses modulated by GBA motifs reported to date are Gβγ-dependent. For example, the most prevalent signaling mechanism modulated by the GBA motifs of GIV or DAPLE is the activation of PI3K in a Gβγ dependent–manner, and other reported pathways, like activation of RhoGEFs, are also under the control of Gβγ (30, 72, 76, 88). If GBA motifs signal primarily *via* Gβγ, what is the functional relevance of their GEF activity in cells? While it is possible that GBA motifs’ GEF activity does not lead to the formation of enough Gα-GTP to be detected by currently available approaches or even to propagate signaling to canonical effectors like adenylyl cyclase, formation of low levels of Gα-GTP upon GBA motif could regulate the directionality and duration of signaling. Since GBA motifs have low affinity for GTP-bound Gα (30, 80), it is conceivable that their GEF activity could serve to facilitate disengagement from G proteins, thereby ensuring finiteness of signaling.

## Transduction of extracellular cues *via* GBA motif–containing proteins

### GBA motifs in signal transduction mediated by receptors that are not GPCRs

**Figure 3:**
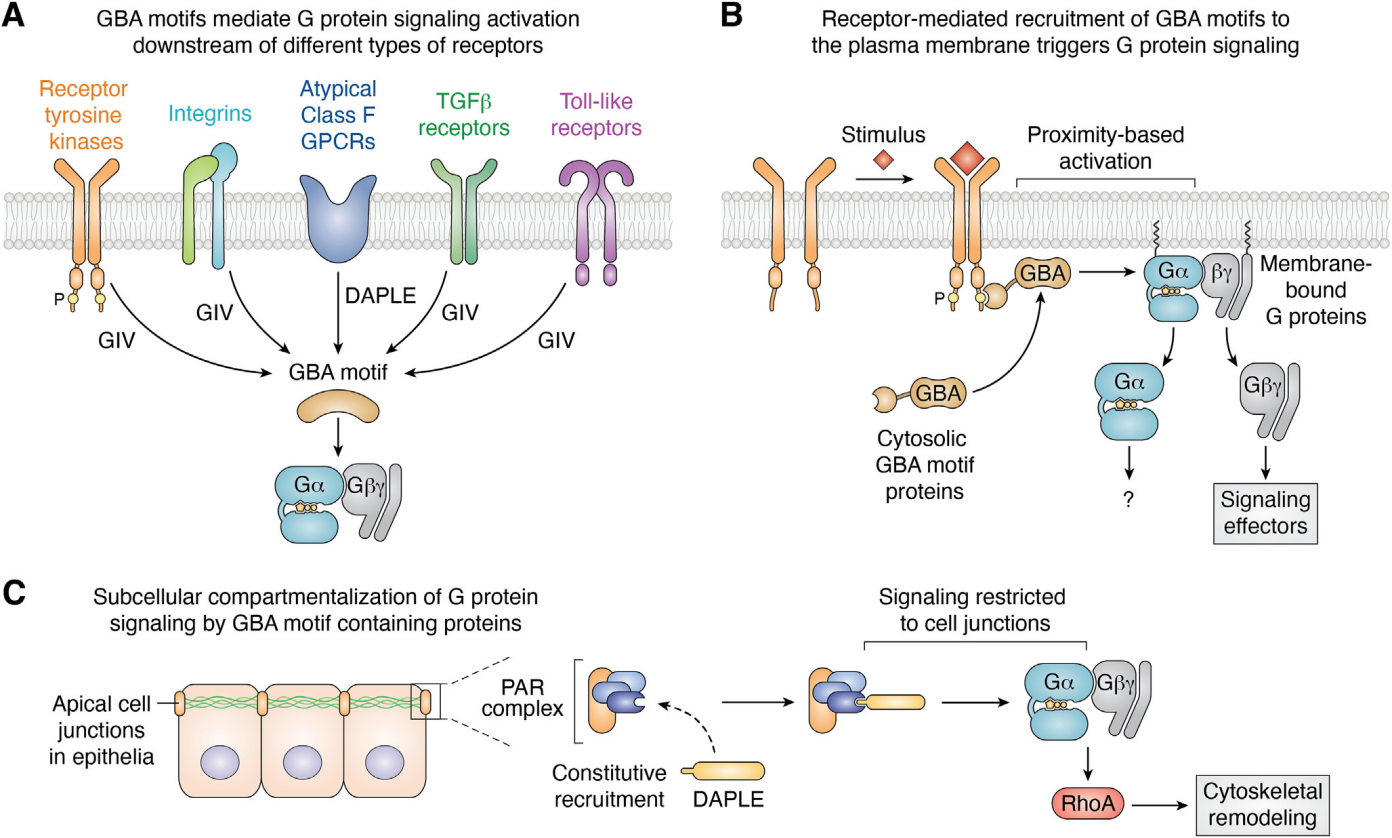
**Signal transduction mechanisms mediated by proteins with a GBA motif.***A*, proteins with a GBA motif propagate signaling through G protein activation upon stimulation of receptors from different families. *B*, recruitment of GBA motifs from the cytosol to the plasma membrane *via* binding to active receptor complexes is sufficient to trigger G protein signaling. G proteins are constitutively attached to membranes, so they cannot be activated by GBA motifs in the cytosol under resting conditions. Once recruited to the proximity of G proteins on membranes, GBA motifs propagate signaling primarily *via* free Gβγ-dependent mechanisms. *C*, DAPLE is constitutively recruited to apical cell junctions in epithelia, where it triggers spatially restricted G protein signaling to remodel the apical actin cytoskeleton. DAPLE, dishevelled-associating protein with a high frequency of leucine residues; GBA, *Gα-binding-and-activating*; GIV, Gα-interacting vesicle-associated protein.

Proteins with a GBA motif are cytoplasmic, so they lack the ability to directly sense extracellular cues that cannot cross the plasma membrane (Fig. 3). Thus, as opposed to the canonical activators of heterotrimeric G proteins, GPCRs, GBA motif–containing proteins must operate under the control of other proteins exposed to the extracellular milieu. From the early studies on GIV, it became evident that its GBA motif had a prominent role in mediating signaling triggered by a large class of receptors different from GPCRs—that is, receptor tyrosine kinases (RTKs) (30, 89, 90). Engineering cells to express GIV variants in which the GBA motif was disabled supported the conclusion that ligands that activate RTKs like insulin or the epidermal growth factor (EGF) propagated signaling to downstream targets like PI3K *via* GBA-mediated G protein regulation (30, 89). Similar conclusions were drawn by engineering cells to express G_i3_ proteins insensitive to GIV (65). A series of papers by Ghosh and colleagues further pinpointed the molecular mechanisms by which GIV mediates RTK signaling and expanded the repertoire of receptors involved, suggesting that G protein activation *via* GIV is a general principle of RTK signaling (91, 92, 93, 94). More specifically, GIV bears an Src homology 2 (SH2)-like domain within its C-terminal region that recognizes autophosphorylated tyrosines in the tails of activated RTKs at the plasma membrane (94). This receptor recruitment event is required for GIV-mediated activation of G protein signaling (93). It has been further proposed that tyrosine phosphorylation of Gαi by RTKs enhances G protein signaling and that this depends on bridging between the receptor and the G protein mediated by GIV (95). These findings are significant because, while the involvement of heterotrimeric G proteins in RTK signaling has been postulated for decades (96, 97, 98, 99, 100, 101, 102, 103), the mechanisms involved have remained puzzling. The detailed insights into how GIV’s GBA motif mediates RTK signaling appear to make it the best understood mechanism by which a major class of receptors like RTKs utilizes signaling machinery typically ascribed to another major class of receptors to transduce signals (*i.e.*, heterotrimeric G proteins used by GPCRs).

RTKs are not the only class of receptors shown to operate *via* GIV and other GBA motif–containing proteins. Other receptors include transforming Growth factor β receptors, toll-like receptors, integrins, or even “atypical” GPCRs of the class F (Frizzled receptors) (29, 72, 104, 105, 106). Of these, the best characterized is the mechanism by which integrins activate G protein signaling *via* GIV. When extracellular matrix components like collagens engage integrins, these receptors recruit GIV to focal adhesions, either directly or *via* interaction with integrin-associated adaptors (106, 107, 108, 109, 110). This recruitment not only leads to activation of G protein signaling *via* GIV’s GBA motif but also results in robust tyrosine phosphorylation of GIV by focal adhesion kinase. Both events must be coincident to propagate integrin signals to the downstream target PI3K (108), which is activated by recruitment of the p85 subunits to phosphorylated GIV and Gβγ released by the action of GIV’s GBA motif (111). The mechanisms by which GIV triggers G protein signaling downstream of other types of receptors is still not well understood.

### What “turns on” GBA motifs so they activate G protein signaling?

If proteins with a GBA motif rely on receptors and potentially other signaling intermediaries to sense and respond to extracellular stimuli, there must be a mechanism that enables their ability to activate G proteins upon receptor stimulation. A common observation across receptor-mediated mechanisms is that GBA motif–containing proteins are recruited from the cytosol to the plasma membrane *via* association with active receptor complexes (89, 93, 106) (Fig. 3). The features of the prototypical example of GIV’s involvement in receptor signaling mediated by RTKs suggested a mechanism of heterotrimeric G protein regulation analogous to that mediated by GEFs for another class of G proteins in this receptor system. A canonical paradigm of RTK signaling is that activation of the small G protein Ras requires recruitment of its GEF, Son of sevenless (SOS) from the cytosol to the plasma membrane *via* the SH2 domain–containing adaptor Grb2 (112, 113). Such recruitment of SOS is not only necessary but also sufficient to serve as the switch that activates Ras based on spatial segregation: Ras is constitutively attached to membranes and remains out of reach from its activator SOS localized in the cytosol under resting conditions, whereas recruitment of SOS to the plasma membrane is sufficient to drive binding to and activation of the G protein. Similarly, GIV (and other GBA proteins) are cytosolic under resting conditions, while their substrate G proteins, G_i_, are permanently attached to membranes (114, 115). Engineering the constitutive attachment of GIV to the plasma membrane or inducing acute recruitment of GIV to the plasma membrane using a chemogenetic approach in the absence of receptor stimulation were sufficient to promote G protein signaling in cells (116). Moreover, signaling of the epidermal growth factor receptor *via* the adaptor Grb2 could be rewired to activate heterotrimeric G proteins by fusing GIV’s GBA motif to the adaptor (116), bolstering the conclusion that membrane recruitment *via* receptor association is sufficient to drive G protein signaling. This membrane recruitment mechanism probably applies broadly to other GBA motif–mediated signaling. For example, in integrin-GIV-G protein signaling or in Frizzled receptor-DAPLE-G protein signaling receptor–mediated recruitment of the G protein activator to cell membranes is also a requirement (72, 106) (Fig. 3). Despite this apparently prominent role of membrane recruitment, it should be noted that the regulation of G proteins by GBA motifs can be further modulated *via* posttranslational modifications. More specifically, phosphorylation of residues adjacent to the GBA motif of GIV have been shown to tune up or down its ability to bind G proteins and regulate downstream signaling (78, 117, 118).

### Theme and variation on the GPR mechanism of DAPLE in cells

The discovery that DAPLE contained a GBA motif was accompanied by the elucidation of a mechanism by which it mediated receptor-initiated responses with similarities to what had been previously observed for GIV in the context of RTK signaling. In cancer cells, DAPLE was shown to promote G protein signaling triggered by Frizzled receptors (72), which belong a class of GPCRs (class F) with unconventional features (119). More specifically, whether Frizzled receptors work as direct G protein activators by exerting GEF activity akin to canonical GPCRs has been debated, although growing evidence supports that they are capable of coupling to G proteins (119, 120, 121, 122). An alternative and nonmutually exclusive model is that DAPLE serves as mediator between Frizzled receptors and G protein activation based on its ability to bind to these receptors and to activate G proteins *via* its GBA motif (72). Similar to what has been observed for GIV-mediated G protein activation in the context of RTKs and integrins (89, 93, 106), DAPLE is recruited from the cytosol to active Frizzled receptors at the plasma membrane, where it promotes G protein signaling primarily by activating pathways that depend on free Gβγ, like activation of PI3K and certain RhoGEFs (72). This mechanism of signaling also seems to operate in the context of noncanonical G protein signaling involved in the organization of the inner ear *in vivo* (123), which has been proposed to rely on polarity cues acting on Frizzleds (*i.e.*, Wnt ligands).

The mechanism described above for DAPLE suggests a conserved theme for the mechanism of activation of G proteins in which GBA motif–containing proteins are diffusely distributed in the cytosol until a receptor-mediated stimulus brings them to the plasma membrane, where their concentration on the two-dimensional plane of the membrane facilitates the interaction with substrate G proteins, which are constitutively membrane-bound (Fig. 3). However, it has become evident that this mechanism is context dependent, as DAPLE expressed in nontransformed epithelial cells, as opposed to cancer cells, does not localize in the cytosol under resting conditions (88, 124, 125, 126, 127). Instead, DAPLE selectively accumulates at apical cell–cell junctions of epithelial cells *via* direct interactions with scaffold proteins like PAR3 or MPDZ that occur *via* DAPLE’s C-terminal PDZ binding motif (88, 124, 128, 129) (Fig. 3). This pattern of subcellular localization of DAPLE is essential for its ability to activate a G protein signaling cascade *via* its GBA motif, which leads to remodeling of the actomyosin cytoskeleton through a Gβγ-p114RhoGEF cascade (88, 124). This represents a variation on the theme described above—that is, while localization at the cell cortex is essential in all cases, this is not triggered by receptor stimulation in the case of DAPLE-G protein signaling in epithelial cells. Instead, this signaling must be constitutive and probably regulated by DAPLE expression levels and/or tissue level remodeling of intercellular contacts. In other words, once the epithelial polarity program leads to the assembly of apical junctional complexes that establish the apico-basal polarity identity of epithelial cells (130), any DAPLE expressed in the cells would be recruited *via* interaction with PDZ domains of junctional scaffolds. The fact that the upregulation of DAPLE expression during embryonic development proceeds in parallel to its recruitment to cell–cell junctions and cytoskeletal remodeling supports this idea (88). The discrete subcellular targeting of DAPLE to apical cell junctions would trigger location-specific activation of G protein signaling in that location, thereby achieving specificity in the responses involved in remodeling the apical cytoskeleton. The latter is reminiscent of the emerging concept of location-bias in canonical GPCR-G protein signaling in which subcellular compartmentalization leads to distinct downstream readouts (131, 132, 133, 134, 135). However, this mechanism of DAPLE-mediated signaling at cell–cell junctions might be subject to additional layers of regulation beyond levels of protein expression, since it has been described that phosphorylation of highly conserved tyrosines adjacent to its C-terminal PDZ binding motif regulate interactions of DAPLE with junctional proteins (128).

An intriguing finding related to this variation on the theme of G protein activation mechanism by GBA motif–containing proteins has been the discovery of an isoform of GIV that bears a PDZ binding motif like the one present in DAPLE, which is also involved in binding to the same cell junctional partners (136). This discovery raises interesting questions about the function of GIV in epithelial cell biology and cancer, as well as on the evolution of GBA proteins of the *CCDC88* family. Regarding the latter, invertebrate *CCDC88* orthologs, which lack the extended C-terminal regions found in GIV and DAPLE, do present a C-terminal PDZ binding motif, suggesting that it might have been retained in different vertebrate orthologs instead of only in DAPLE as initially suspected. As for the role of GIV in epithelial cell biology and cancer, some apparently contradictory observations could be explained by the existence of different isoforms. For example, GIV has been shown to be important to maintain the nontransformed phenotype of epithelial cells by facilitating the maintenance of proper cell–cell junctions *via* G protein regulation (137, 138), whereas it also facilitates the acquisition of invasive phenotypes of cancer cells (*e.g.*, cell migration) associated to the loss of epithelial phenotype (30, 75, 139). These contrasting roles might be explained by different roles of isoforms with and without a PDZ binding motif (136).

## Physiological functions regulated by GBA motifs and pathologies associated with their dysregulation

### GBA motif–mediated signaling in cancer

Among GBA motif–containing proteins, GIV and DAPLE are the ones with best characterized cell biological functions. Interestingly, the role of their GBA motifs in regulating cell functions were not initially identified in physiological contexts but in controlling abnormal cell signaling in cancer. For example, soon after the first identification of a GBA motif in GIV (30), it became clear that disabling its ability to regulate G proteins by specifically disrupting the GBA motif blunted proinvasive traits like cell migration and related signaling pathways in cancer cells (30, 65, 75, 76, 89). This mechanism is specifically enabled in invasive cancer cells because of GIV overexpression. Compared to nontransformed epithelial cells or nonmetastatic tumor cells, GIV expression is upregulated in metastatic cancer cells (139, 140), possibly *via* STAT3 among other transcription factors (141), which in turn enables the engagement of G proteins *via* its GBA motif and subsequent enhancement of proinvasive signaling mechanisms. This correlation between metastasis and GIV upregulation is also observed in human tumors *in situ*. For example, an initial study with a small cohort of colorectal cancer patients revealed higher expression of GIV in higher stage, metastatic tumors (139). Moreover, expression of GIV in lower stage, nonmetastatic tumors was inversely correlated with patient survival (139). Since metastasis is the cause of >90% of cancer deaths, a reasonable explanation for this observation is that GIV expression favors the progression of cancer toward metastasis, resulting in a higher mortality. The conclusions of this seminal study linking GIV to increased metastasis and diminished patient survival has been extensively validated and expanded to several other types of cancer and with much larger cohorts, including some with hundreds of cancer patients (141, 142, 143, 144, 145, 146, 147, 148, 149, 150, 151, 152, 153, 154, 155, 156, 157, 158). Overall, the key role of GIV’s GBA motif in regulating different behaviors of cancer cells that facilitate invasiveness (30, 65, 75, 76, 89, 159, 160, 161) make inhibiting it an attractive target to develop novel antimetastatic therapeutic approaches.

It was also in the context of cancer where the role of the GBA motif of DAPLE in disease became apparent first. This was made possible again by specifically disabling the GBA motif of DAPLE through site-directed mutagenesis in cancer cell lines (72). However, the expression pattern and role of DAPLE during cancer progression was more complex than in the case of GIV. Much like what had been observed with GIV, activation of G protein signaling by DAPLE favors the proinvasive traits of metastatic cancer cells (72). Further expanding on the similarities with GIV, this role of DAPLE as a driver of signaling programs that promote aggressiveness has been linked to the ability to propagate signaling initiated by RTKs in head and neck cancers (162). However, DAPLE is also expressed in nontransformed epithelial cells, where it seems to work as a tumor suppressor by preventing the initial process of transformation (72). In agreement with these observations, the expression of DAPLE in tumors *in situ* at different stages of colorectal cancer progression also shows a biphasic regulation—DAPLE expression is first lost in the transition from nontransformed polyps to localized carcinomas, but later upregulated in metastatic tumors (72). Moreover, it has also been described that two different isoforms of DAPLE contribute differently to the progression of cancer at different stages (163). The role of DAPLE as a tumor suppressor is not surprising given that it has been convincingly shown that in normal epithelial cells DAPLE localizes to apical cell–cell junctions (88, 125, 126, 128, 129, 164), where it controls cellular architecture and function *via* G protein regulation (88, 124). It is reasonable to believe that when DAPLE is lost in this context, cytoarchitectural changes in epithelial cells make them more prone to transformation.

### GBA motif–mediated signaling in physiology and development

While the work on GIV and DAPLE summarized above established that they regulate G protein-dependent signal transduction in cultured cells (30, 72, 92, 93, 106, 116, 165) and that this mechanism is dysregulated in cancer (29, 72, 75, 162, 166, 167), direct evidence *in vivo* for a physiological process controlled by GBA motifs was still lacking. The first evidence supporting the role of GBA motif–mediated signaling *in vivo* came through the investigation of DAPLE in normal embryonic development of the nervous system (88). In *Xenopus* and zebrafish embryos, DAPLE is specifically induced during neurulation and is required for the formation of the neural tube by driving the bending of the neuroepithelium (88). This is achieved by activating a GBA motif–dependent signaling pathway in which free Gβγ activates RhoA-dependent actomyosin contractility *via* p114RhoGEF (88). This results in the contraction of the actomyosin meshwork that is anchored at apical cell–cell junctions (168). Interestingly, these findings established that DAPLE is a tissue-specific G protein activator in the context of apical cell constriction during embryo morphogenesis in vertebrates, a function that had been ascribed to GPCRs in invertebrates without clear counterparts in vertebrates (169). The latter suggests that receptor-independent regulation of G protein mechanisms in this context might have appeared as an evolutionary specialization in vertebrates.

Defining the role of DAPLE-mediated G protein signaling in normal embryonic development also provided insights into the molecular basis of human disease. DAPLE has been recently described to be mutated in human patients with nonsyndromic congenital hydrocephalus (170, 171, 172, 173), a neurodevelopmental disease that manifests as a frequent birth defect (∼1:1000) characterized by the enlargement of brain ventricles due to fluid accumulation (174, 175, 176) and that is associated with high morbidity and mortality rates (176, 177, 178, 179) leading to a huge medical cost (∼$2 billion per year) (180, 181, 182). Some of the DAPLE mutations found in hydrocephalus result in the loss of its GBA motif, which together with the requirement of DAPLE in shaping the embryonic brain suggests a relationship between dysregulated GBA motif–mediated signaling and hydrocephaly.

Although the physiological functions specifically controlled by GIV *via* its GBA motif have not been defined, there is a curious parallelism with the role of DAPLE in neurodevelopment. More specifically, GIV is mutated in yet another human neurodevelopmental disorder that causes profound mental disability (183, 184)—that is, progressive encephalopathy with edema, hypsarrhythmia, and optic atrophy–like syndrome. In fact, GIV expression is highest in the brain, along with testis, across primary mouse tissues (185), and the main phenotype of mice lacking GIV is brain developmental defects that mimic the progressive encephalopathy with edema, hypsarrhythmia, and optic atrophy–like syndrome (183, 184), which results in premature death several weeks after birth in mice (186, 187, 188). While it is not known if G protein regulation by GIV plays a role in this context, it is interesting that loss of either GIV or DAPLE results in nonlethal embryonic neurodevelopmental defects, pointing to a possible functional redundancy in brain morphogenesis.

DAPLE-mediated G protein signaling may have other developmental roles beyond neurodevelopment, as it has been shown that loss of DAPLE causes deafness in mice (189), which is most likely related to the role of DAPLE in the development of proper architecture of epithelial cells in this tissue (126, 127). It is well established that heterotrimeric G proteins are involved in the morphogenesis of specialized epithelial cells in the inner ear responsible for auditory sensation but that this mechanism does not rely primarily on canonical GPCR regulation (126, 127, 190, 191, 192, 193, 194, 195, 196). DAPLE has been proposed as one of the critical regulators of Gαi subunits in this context, which appears to work coordinately with other non-GPCR regulators of G proteins like GoLoco-motif containing proteins and RGS proteins to form an alternative G protein cycle (126, 127).

### Involvement of GBA motif signaling in other diseases

GIV has been involved in a number of human diseases in addition to cancer. As mentioned above, GIV expression in normal tissues is highest in brain and testis. It has been recently reported that GIV deficiency correlates with male infertility in humans, and that GIV’s ability to regulate G proteins *via* its GBA motif has a pivotal role in sperm capacitation (197). It is therefore conceivable that specifically disrupting GIV-G protein coupling could serve as a male contraceptive. In kidney failure (nephrotic syndrome), GIV expression is upregulated as an adaptive response to injury, leading to a protective effect through G protein–dependent activation of prosurvival pathways triggered by growth factor receptors (198). Lopez-Sanchez *et al.* also demonstrated a role for GIV in liver fibrosis that is mediated by its GBA motif (105). More specifically, GIV is upregulated in hepatic stellate cells in mouse models of liver fibrosis and in patients with liver fibrosis. GIV-mediated G protein signaling in hepatic stellate cells, which are central in driving fibrotic transformation in the liver, enhances fibrotic pathways while dampening antifibrotic ones when it is upregulated (105).

## Experimental therapeutics approaches to target and manipulate GBA motif–mediated signaling

The prominent role of GBA motif–mediated signaling in various diseases, as described above, makes targeting it an attractive goal to envision new therapeutics. Moreover, developing new means to target and manipulate GBA motif–dependent signaling as research tools also holds the promise of accelerating the investigation of fundamental aspects of this mechanism of G protein regulation that remains poorly understood. Efforts to develop approaches for the targeted manipulation of signaling mediated by GBA motifs summarized below has been facilitated by the molecular and structural characterization of the engagement of GBA motifs with Gα subunits summarized in preceding sections.

### GBA motif mimics

**Figure 4:**
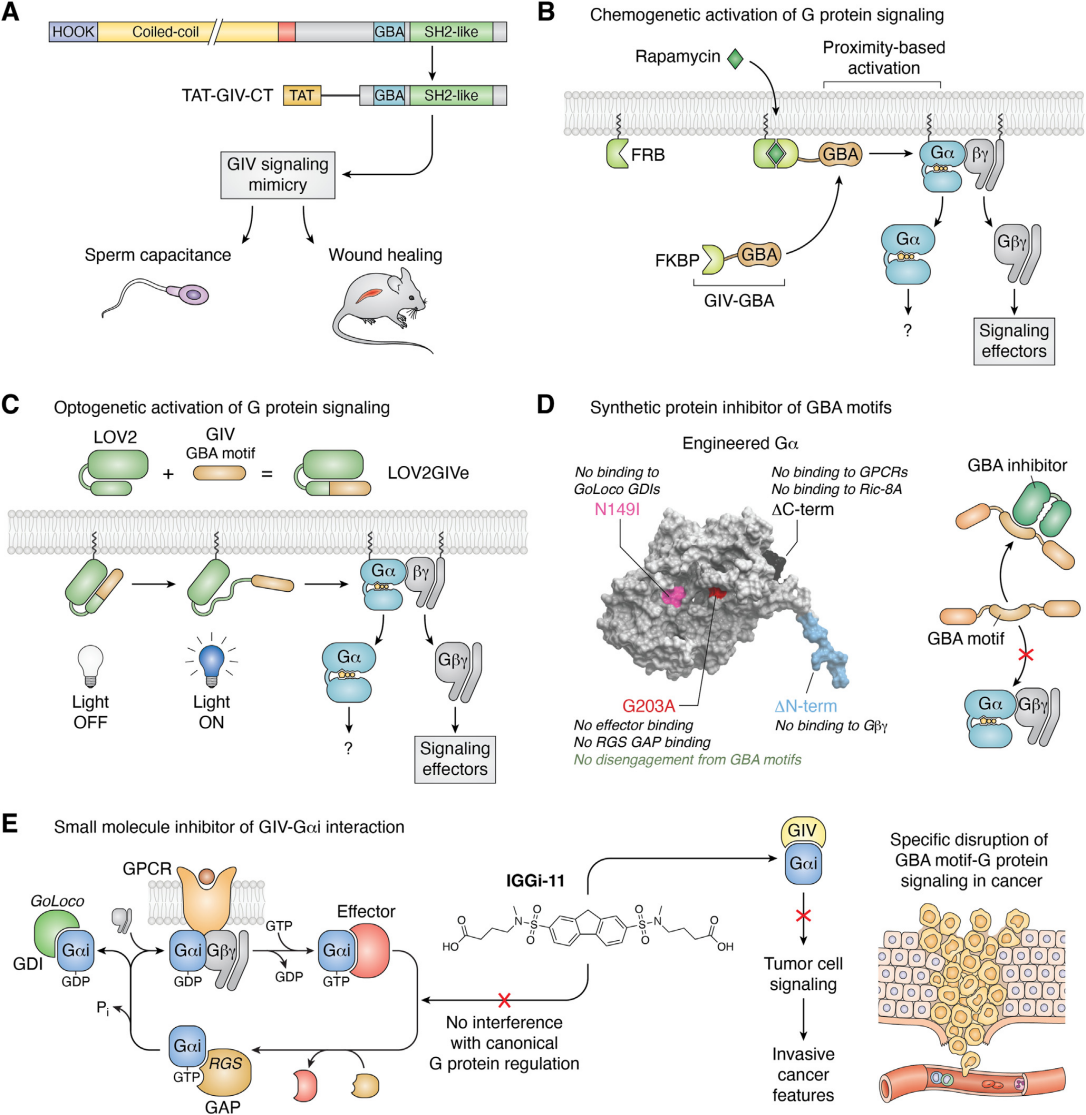
**Tools to leverage or investigate signaling mediated by GBA motifs.***A*, TAT-fused C-terminal region of GIV (TAT-GIV-CT) can be delivered to cells as a cell-penetrating protein that mimics the signaling activity of GIV. TAT-GIV-CT has been shown to increase human sperm capacitance and skin wound healing in mice. *B*, chemogenetic control of the subcellular localization of GBA motifs allows to control G protein signaling activation in cells. *C*, fusing a GBA motif to the light-sensitive LOV2 domain of *Avena sativa* (LOV2GIVe) allows for optogenetic control of G protein signaling in cells. *D*, an engineered Gα subunit that constitutively binds to GBA motifs but does not bind to other G protein regulators and effectors serves as genetically encoded “GBA inhibitor” (GBAi). *E*, a small molecule that binds to Gαi named IGGi-11 competitively inhibits signaling mediated by GIV–Gαi complexes in cancer without affecting canonical mechanism of G protein signaling mediated by GPCR and downstream pathway effectors and regulators. GBA, *Gα-binding-and-activating*; GBAi, GBA inhibitor; GIV, Gα-interacting vesicle-associated protein; GPCR, G protein–coupled receptor; IGGHi-11, inhibitor of the GIV–Gαi interaction; TAT, transactivator of transcription.

The first successful case of targeting signaling mediated by GBA motifs came through the development of cell penetrating proteins that mimicked their actions. More specifically, the team led by Ghosh created constructs in which the C-terminal region of GIV is fused to a sequence derived from the transactivator of transcription of the HIV known to facilitate cellular uptake into the cytoplasm (92) (Fig. 4*A*). The fragment of GIV used in this construct contained not only the GBA motif but also other elements involved in GIV-mediated signaling, like its SH2-like domain that allows binding to active RTKs. Purified versions of this protein construct were efficiently taken up by cells when added to the culture medium and faithfully recapitulated the functions of full-length GIV in regulating signaling (92). This approach has been successfully implemented to facilitate wound repair (92), enhance sperm capacitance (197), modulate immune responses (104), or mimic profibrotic, and prometastatic phenotypes associated with GIV’s GBA motif function (92, 105).

More recently, there have been several studies aimed at identifying peptide modulators of heterotrimeric G proteins that have converged into the identification of GBA-like sequences. For example, Nubbemeyer and colleagues described peptides similar in sequence to GBA motifs that also mimic the dual ability to promote nucleotide exchange on Gαi while inhibiting it on Gαs (199). These sequences are also reminiscent of the synthetic KB-752 peptide that led to the initial identification of a GBA motif in GIV (64) and of other directed evolution screens that have identified Gα binding peptides (200). The peptides identified by Nubbemeyer and colleagues were fused to cell penetrating peptides, which led to modulation of G protein signaling when administered to cells (199). Similarly, a collaborative study by the teams led by Suga and Shokat has recently found GBA-like peptides by screening a large library of macrocyclic peptides (201). More specifically, a cyclic peptide that binds selectively to GDP-bound Gαs, named GD20, not only conformed with the consensus of the GBA motif sequence but also engaged with the G protein using the same overall pose and *via* equivalent amino acid side chains according to the structure of the Gαs-GD20 structure that was resolved (201). Cell penetrating versions of the GD20 peptide also led to dissociation of Gβγ from Gαs, much like it has been described for GBA motifs (87). In addition to providing useful research tools, these multiple, independent studies converging on GBA-like peptides highlight that the consensus GBA sequence is a privileged motif for the engagement of Gα subunits.

A related approach has consisted of generating genetically encoded constructs based on GIV’s GBA motif that allow to manipulate G protein signaling with high precision. For example, a chemogenetic approach leveraging chemical dimerizers allows to trigger G protein responses by rapidly translocating GBA motifs from the cytosol to the plasma membrane (73, 87, 116) (Fig. 4*B*). Another genetically encoded tool, named LOV2GIVe, also permits to control GBA motif–mediated activation of G proteins with light (202) (Fig. 4*C*). This optogenetic tool is based on “caging” the GBA motif of GIV by fusion to the light-sensitive LOV2 domain of *Avena sativa*, which is then released upon blue light exposure to allow access to the G protein target.

### Engineered synthetic GBA motif inhibitor protein

While mimicking GBA motifs has useful applications in the context of manipulating G protein signaling, approaches to specifically block the action of GBA motifs are more useful to decipher their biological roles and/or correct abnormalities caused by their excessive activity frequently associated with human disease (*vide supra*). To this end, the synthetic protein named GBA inhibitor (GBAi) was developed (165). GBAi is a construct rationally engineered from a Gαi scaffold by removing the ability of the G protein to interact with any known interactor, including Gβγ subunits and GPCRs among other regulators and effectors, while favoring constitutive high affinity binding to GBA motifs (165). Practically, GBAi can only bind to GBA motifs and it cannot disengage from them regardless of nucleotide loading status, resulting in competitive displacement of GBA motifs from Gα subunits in cells (Fig. 4*D*). When implemented as a genetically encoded construct, GBAi efficiently suppresses receptor-mediated signaling and phenotypes mediated by GBA motif–containing proteins both in cultured cells and in whole organisms *in vivo* but without interfering with canonical G protein signaling mechanisms triggered by GPCRs (165).

### Small molecule targeting of GBA motif–mediated signaling

Metastasis is the cause of >90% of cancer-related deaths but there are very limited therapeutic options for patients bearing cancers of such advanced stages. Given this important medical unmet need and the fact that dysregulated G protein signaling GIV favors the progression of cancer toward metastasis, the idea of targeting GBA motif–mediated signaling was postulated early on (30). This idea was further bolstered by the fact that the expression of GIV in normal tissues is largely restricted to brain and testis (185), suggesting that it might be possible to target the disease-specific mechanism without having overt side effects. However, the main challenge of targeting GBA motif–mediated signaling is that it relies on disrupting a protein–protein interaction, which is traditionally considered a difficult target. A prerequisite to pursue protein–protein interaction targets is to have a good understanding of the structural basis for it and robust assays to monitor them. The rapid progress on characterizing GIV-Gαi binding through various complementary approaches described above set the stage to assess the experimental tractability of this protein–protein interface’s “druggability”—that is, empirically testing if chemical compounds can disrupt the protein–protein interaction. In a first pilot assessment combining computational and wet lab experiments (203), a previously described compound known to bind Gαi emerged as an inhibitor of the GIV–Gαi interaction (204). While the compound, NF023, was of limited utility because of its lack of specificity, among other reasons, it supported the idea that the GIV-Gαi interface was a tractable druggable target for different reasons. In addition to providing a *de facto* demonstration for the feasibility of breaking up the interaction between GIV and G proteins by a chemical compound, the observations supported that the mechanism of action of NF023 was compatible with prior structural knowledge because its binding site on Gαi overlapped with that of GIV, yet it did not disrupt binding of other Gα interactors like Gβγ (203). These findings motivated a screen of a larger collection of ∼200,000 compounds, which resulted in the identification and validation of one compound with desirable properties of efficacy and specificity (205). More specifically, this compound, named inhibitor of the GIV–Gαi interaction 11 (IGGi-11), binds with micromolar affinity to the GIV binding region of Gαi, thereby precluding binding of the G protein regulator (Fig. 4*D*). Despite binding to the G protein, potential on-target but undesired effects were thoroughly ruled out. Essentially, IGGi-11 had no effect on the ability of Gαi to bind and hydrolyze nucleotides, or to bind to Gβγ, or to become activated by GPCRs, or to interact with effectors like adenylyl cyclase or with regulators like GDIs and GAPs. A cell permeable analog named IGGi-11me also displayed favorable properties in signaling assays. For example, it disrupted signaling mediated by GIV in cancer cells without affecting canonical GPCR-mediated signaling independent of GIV in multiple cell lines and assay formats, including second messengers (cAMP) and kinase cascades (Akt). Moreover, the compound also blunted tumor cell migration and cell growth under tumor-like conditions, like cultures on Matrigel or after subcutaneous implantation of cancer cells in mice post-IGGi-11me treatment *ex vivo* (205). The specificity of the effects of IGGi-11me on the intended target was supported by the loss of efficacy in signaling and migration assays in cells depleted of GIV (205). While IGGi-11me is far from being useful for therapeutics due to its modest potency and pharmacokinetic properties, it provides the proof of principle for the targeting of G proteins to disable atypical mechanisms of regulation. Although it will be required to generate improved IGGi-11 analogs to gain therapeutic value, the specificity of the existing compound could be leveraged as a tool to characterize signaling mechanisms mediated by GBA motifs. It should be noted that IGGi-11 is likely to block signaling not only mediated by GIV but also by other GBA motif–containing proteins given that the structural and molecular basis for their binding to Gα subunits is very similar (28, 73, 80). In fact, IGGi-11 disrupts the binding of DAPLE to Gαi and cells treated with IGGi-11me phenocopy cells depleted of DAPLE. A small molecule tool like IGGi-11 is a good complement to genetically encoded tools like GBAi (described above) to facilitate the investigation of noncanonical G protein signaling with greater convenience than the standard to date—that is, engineering cells expressing GBA-deficient versions of regulators of this class.

## Conclusions and future perspective

This review has summarized progress made on characterizing a mechanism of G protein signaling activation independent of GPCRs across scales of biological organization, from the molecular level, to phenotypes in cells, to physiological and pathological consequences at the organismal level, to return to the molecular level by covering related pharmacology and experimental therapeutics. The focus has been on a particular type of regulators that are grouped as the same class due to the presence of a GBA motif. Progress has been relatively fast, especially considering the limited number of research teams that have actively engaged in the study of this class of regulators. While the reasons for the limited engagement are unclear, one potential challenge is the experimental tractability of the problem. For example, providing definitive evidence for the role of the GPR function of a given GBA motif–containing protein requires methods more sophisticated than simply knocking down, knocking out, or overexpressing the protein of interest because these proteins have functions in addition to activating G protein signaling. Instead, it is typically required to replace WT proteins in cells with counterparts in which the GPR function is specifically disabled. Moreover, the archetypes of this family, GIV and DAPLE, are encoded by complementary DNAs of >5.5 Kb, which makes genetic manipulation or delivery cumbersome and difficult (*e.g.*, extensive sequencing validation is required for constructs and viral packaging becomes inefficient). To stimulate further engagement with this area of investigation, this review has also highlighted tools developed so far to characterize signaling mediated by GBA motifs with more ease and convenience. These tools may facilitate addressing several of the existing open questions. For example, we still do not know how many proteins bear a functional GBA motif, and for those that we know, we still have limited information about their biological functions. Leveraging genetically encoded and chemical tools described in this review could allow advancing more rapidly in defining how G protein regulation by GBA motifs affects signaling, similar to how pertussis toxin has been historically used to study G_i/o_ protein regulation by GPCRs. Another important question will be to address the interplay of GBA proteins with other non-GPCR regulators, like GAPs, GDIs, and other GEFs, in controlling G protein function. While there is evidence that this type of interplay among non-GPCR regulators exists to control important biological processes (127, 206, 207, 208), we have just barely begun to scratch the surface of how alternative G protein cycles may operate. Similarly, how these GPCR-independent mechanisms intersect with and influence GPCR-mediated signaling is still poorly understood (86, 87) but could have a profound impact on determining context-dependent regulation of receptor biology and pharmacology. Moreover, gaining deeper insight into how GBA-dependent mechanisms of G protein regulation in disease and their potential pharmacological modulation may also spur renewed interest in investigating and targeting novel components of GPCR pathways beyond the widely pursued receptors.

## Conflict of interest

The authors declare that they have no conflicts of interest with the contents of this article.
